# Impact of Engineered Carbon Nanodiamonds on the Collapse Mechanism of Model Lung Surfactant Monolayers at the Air-Water Interface

**DOI:** 10.3390/molecules25030714

**Published:** 2020-02-07

**Authors:** Aishik Chakraborty, Amanda Hertel, Hayley Ditmars, Prajnaparamita Dhar

**Affiliations:** Department of Chemical and Petroleum Engineering, The University of Kansas, Lawrence, KS 66045, USA; ashikchakraborty.12@gmail.com (A.C.); amandakay@ku.edu (A.H.); hditmars@pandapediatric.com (H.D.)

**Keywords:** monolayer collapse, engineered nanoparticle, surface pressure-area isotherm, folding, lung surfactants

## Abstract

Understanding interactions between inhaled nanoparticles and lung surfactants (LS) present at the air-water interface in the lung, is critical to assessing the toxicity of these nanoparticles. Specifically, in this work, we assess the impact of engineered carbon nanoparticles (ECN) on the ability of healthy LS to undergo reversible collapse, which is essential for proper functioning of LS. Using a Langmuir trough, multiple compression-expansion cycles are performed to assess changes in the surface pressure vs. area isotherms with time and continuous cyclic compression-expansion. Further, theoretical analysis of the isotherms is used to calculate the ability of these lipid systems to retain material during monolayer collapse, due to interactions with ECNs. These results are complemented with fluorescence images of alterations in collapse mechanisms in these monolayer films. Four different model phospholipid systems, that mimic the major compositions of LS, are used in this study. Together, our results show that the ECN does not impact the mechanism of collapse. However, the ability to retain material at the interface during monolayer collapse, as well as re-incorporation of material after a compression-expansion cycle is altered to varying extent by ECNs and depends on the composition of the lipid mixtures.

## 1. Introduction

Langmuir monolayers at the air-water interface, demonstrate several 2-D phases and phase transitions, ranging from gas-like phase to more condensed phases, when compressed laterally [1]. However, if the monolayer film is compressed beyond its stability limit, a transition from 2-D to 3-D structure occurs. This transition from 2-D to 3-D, referred to as monolayer collapse, occurs at a constant surface pressure. Monolayer collapse has been a subject of interest, especially for biological lung surfactant mixtures that are present at the air-water interface in alveoli of lungs and helps reduce the work of breathing by maintaining near zero surface tension during compression, while maintaining a stable film during monolayer collapse. There are several different mechanisms of collapse, including buckling, budding and vesiculation, as reviewed in detail by Lee [2]. Further, during cyclic compression and expansion, monolayers can collapse either reversibly or irreversibly. Of particular interest, in lung surfactant films, is the buckling mechanism of collapse. Buckling of the film is characterized by formation of a 3-D structure called folds that forms invaginations into the subphase, retaining the molecules close to the surface. Folds “unfold” upon expansion, allowing the collapsed material to be re-incorporated into the monolayer, thus making the collapse reversible. These folds are formed perpendicular to the axis of compression (or parallel to the barriers used for compression), and are often formed across the entire width of the Langmuir trough. On the other hand, complex amphiphilic mixtures, when compressed, can also be prone to a rejection of molecules from the surface before reaching the limiting area of compression. Collapse following this path is deemed irreversible. In this case, a loss in the material at the interface is inevitable. Therefore, it is believed that proper lung surfactant function requires formation of folds that will allow reversible collapse.

Of particular interest to public health and safety is how inhaled particles, such as nanoparticles and pollutants in the air, effect lung surfactant function. The lung surfactants act as barrier against particles that are small enough to deposit inside the alveoli. Several factors including particle shape, density, the size of the inhaled material, as well as the health status and mode of breathing, play key roles in the fate of such particles inside the lungs [3]. The size determines the physical mechanism by which the particles deposit in the lungs. Particles that are larger than 5 microns usually deposit through inertial impaction or gravitational sedimentation (1–8 microns), whereas, particles smaller than 500 nm deposit in the lungs through Brownian diffusion [4]. Thus, depending on the size, the particles can end up either in the upper respiratory tract or even reach the alveoli. Larger particles, usually within 10 to 20 microns, mostly deposit in the upper respiratory tract, whereas, particles as big as 5 microns may deposit in the alveoli [5]. In case of evaluating the toxicity of nanomaterials, the smaller range is of particular interest as the nanoparticles fall within this limit. 

With the advancement in nanotechnology, engineered nanoparticles have been gaining significant grounds in different areas including biomedicine [6,7,8,9]. The small size of the particles as well as relative ease in surface-tunability make these nanoparticles suitable vehicles for targeted drug delivery amongst other biomedical applications [10,11]. Therefore, it is highly likely for nanoparticles to either intentionally or unintentionally enter human bodies, and it is necessary to evaluate the compatibility and the toxicity of the nanoparticles when interacting with various physiological components. In this regard, the respiratory tract is one common route for the entry of the nanoparticles. The small size allows the particles to reach the depths of the alveoli [12]. Upon entering the alveoli, nanoparticles then interact with LS monolayer. Research has shown the impact of size, hydrophobicity, and concentration of different nanoparticles on the functioning of surfactants [13,14,15,16,17,18,19,20]. Many of these studies monitor changes in the surface pressure vs. area isotherms due to incorporation of nanoparticles, thus focusing on the thermodynamic aspects of the changes induced by nanoparticles on model membranes. More recently, some of these studies have also coupled studies focused on changes in the surface pressure with studies monitoring nanoparticle induced changes in the morphology of these films. Such studies have presented new information about the impact of nanoparticles on model lipid monolayers [13,14,15,16,21]. For example, Tatur et. al., showed that even though the isotherms of different model surfactants are not affected by their interaction with hydrophobic gold nanoparticles, the morphology at the air-water interface is predominantly altered in the case of the particle exposure [21]. Thus, in addition to the surface pressure-area isotherms, the surface morphology should be carefully studied while assessing the behavior of the nanoparticles. Similarly, previous work from our lab has shown that the actual composition of the lipid mixture used can also lead to differences in interactions with nanoparticles [22]. However, the impact of nanoparticles on collapse mechanism in phospholipid monolayers is currently not well understood and has not been studied in detail. Understanding how nanoparticles impact the mechanisms of collapse in lung surfactant monolayers is an important issue that should be addressed and is the main focus of this paper. We are particularly interested in studying the impact of carbon-based nanoparticles, specifically engineered carbon nanodiamonds (ECNs), on the mechanisms of monolayer collapse in phospholipid monolayers. 

Studies have shown that the chemistry of the surfactant mixtures (phospholipid combinations and presence of LS proteins or their synthetic analogs) enable fold formation during compression and reincorporation of material during expansion cycles [23,24,25]. On the other hand, our previous work, focused on ECN induced changes in lipid domain packing in the LE-LC regions has shown that differences in lipid-ECN interactions are modulated by other lipid headgroup charge and tail saturation [22]. Specifically, we observed that at lower surface pressures, the anionic ECNs behave as line active species when interacting with zwitterionic phospholipids. But, in the presence of anionic phospholipids, electrostatic repulsion plays a greater role. We hypothesize that ECN induced changes in mechanisms of monolayer collapse should also depend on the composition of the lipid mixtures used. Therefore, in this work, we focus on ECN induced changes in the reversible and irreversible collapse of model lipid membranes using four different lipid mixtures that reflect the lipid headgroups and tail saturations commonly seen in lung surfactants. 

While native surfactants are made up of 90% by weight lipids and 10% by weight proteins, in this work, we only focus on the interactions between the phospholipids and the ECN, in the absence of proteins [26,27]. Zwitterionic, disaturated dipalmitoylphosphatidylcholine (DPPC) is the most abundant phospholipid present in biological lung surfactants and is often used either as the major component or on its own to evaluate the efficacy of novel synthetic lung surfactants [28]. Therefore, in this study DPPC is used as the major lipid component (making up 70% by weight of the lipid mixture). Additionally, unsaturated phosphatidylcholine as well as negatively charged, saturated and unsaturated phosphatidylglycerol are found in native LS mixtures, and are often used in synthetic LS mixtures. Therefore, in this study lipid mixtures containing saturated and unsaturated phosphatidylglycerol were also used at 30 wt.% to study the interaction of ECNs with LS mixtures having a net negative charge reflecting the overall charge of LS monolayers. Lipid mixtures containing DPPC with 30 wt.% unsaturated phosphatidylcholine as well as saturated phosphatidyl-etholamine were used as zwitterionic lipid mixtures. Analysis of cyclic compression expansion surface-pressure isotherms, combined with fluorescence images of the surface of the monolayer during monolayer collapse are together used to arrive at conclusions regarding the impact of ECNs on the mechanisms of monolayer collapse, and the role of lipid headgroup charge and tail saturation on ECN-induced changes in monolayer collapse. Our results together also present ECN-induced changes in the ability of different LS model mixtures to reincorporate material during multiple compression-expansion cycles.

## 2. Results

### 2.1. Isotherms and Area under the Curve of DPPC:POPG

**Figure 1:**
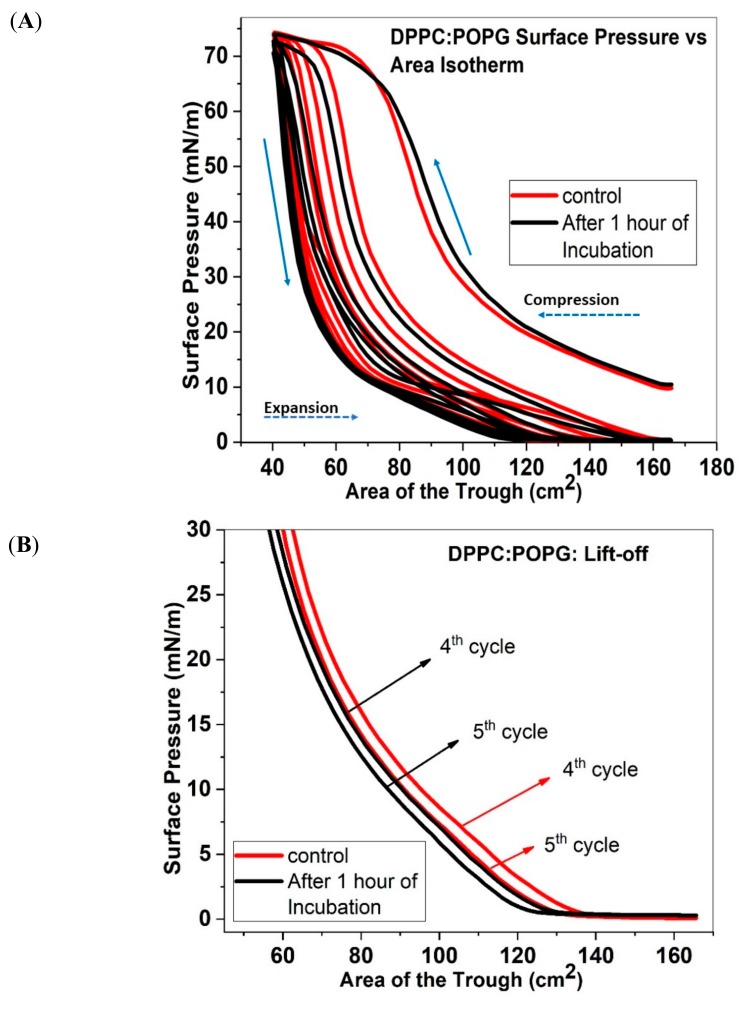

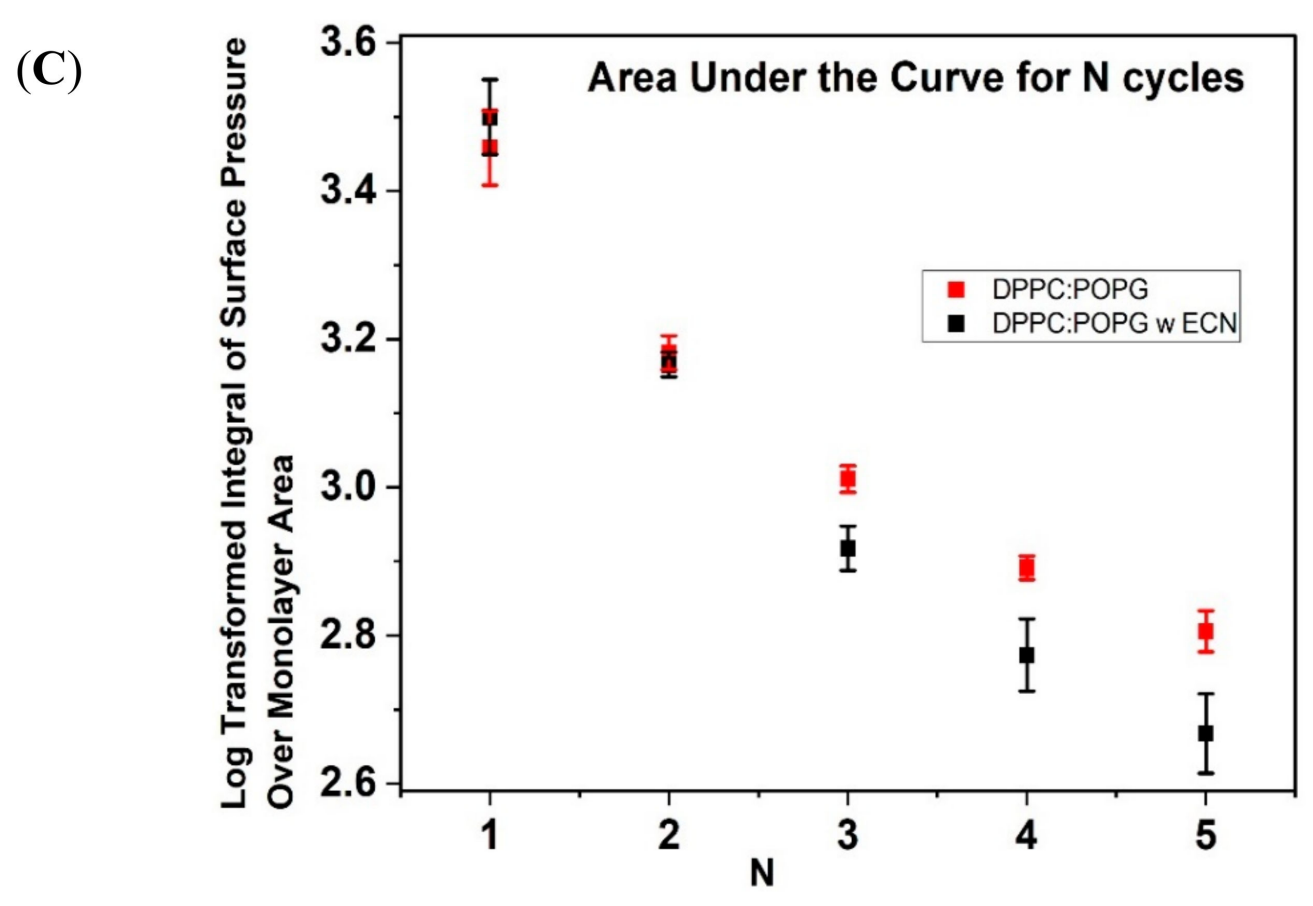
(**A**) Surface Pressure versus area of the trough isotherms for DPPC:POPG (7:3) without (control, red solid line) and with 1 wt.% ECN (black solid line). All samples were compressed and expanded five times. The solid arrows point to the direction of compression curve and the expansion curve on the isotherms. The dashed arrow on the other hand shows the direction of advancement of the compression/expansion cycles. All data has been represented as the mean of 3 samples. (**B**) The initial part of the 4th and 5th compression cycles of DPPC:POPG monolayers with (black lines) and without (red lines) the ECN, showing the lift off area for each compression cycle. (**C**) Area under the curve as a function of compression/expansion cycle for DPPC:POPG (7:3) without (control, red square) and with 1 wt.% ECN (black square). A sample size of three was used for finding the log-transformed mean and standard deviation of the sample.

Figure 1A shows the quasi-static surface pressure versus area of the trough isotherm of DPPC:POPG monolayer without (red solid line) and with (black solid line) 1 wt% ECN. Mathematically, surface pressure can be written as shown in Equation (1) below:

Π = γ_o_ − γ (1)

where, γ_o_ is the surface tension of water and γ is the surface tension of monolayer. 

Initially, at higher area of exposure, the molecules are spread far apart. As the film is laterally compressed, the molecules come closer together and there is an increase in the surface pressure. This leads the monolayer to transition from the gaseous phase to the LE phase. In the case of DPPC:POPG isotherms, we observe that the monolayer is in the LE phase around 10 mN/m of surface pressure. Beyond this Π, the phase coexistence region appears, where the monolayer consists of both LE and LC phase. As the surface area is further reduced, Π increases sharply until 65 mN/m. After this pressure point, the monolayer undergoes a final collapse, encountering a decreasing slope in the isotherm, and eventually reaching pressures of around 72 mN/m. Once the monolayer is fully compressed, the expansion phase begins, and the surface pressure drops rapidly without any notable change in the surface area. The drop continues around 15 mN/m after which the decrease in slope becomes gradual. Here, the expansion curve follows a different path from the compression curve, and therefore, the isotherm displays hysteresis. Multiple reasons have been considered to explain this hysteresis in the Π–A isotherm of lung surfactant monolayers [29]. Alteration in the compositional ratio after expansion may lead to hysteresis. Also, ejection of material from the surface that fails to or slowly reincorporates with expansion may also contribute towards hysteresis. Further shift in the Π–A compression/expansion isotherm is because of a loss in material from the surface. As shown in Figure 1A, during the first compression/expansion cycle, DPPC/POPG isotherms exposed to 1 wt% ECN shifts negligibly to higher molecular areas. Furthermore, the slope of the curve remains identical to that of the control. However, with repeated compression/expansion cycles, the isotherm shifts to lower areas of the trough. As a result, after the end of the first compression-expansion cycle, the monolayer is in gas phase. Upon further compression, the monolayer can transition from gaseous to the LE phase. This transition is termed “lift off”. Figure 1B also shows that between the start of the 4th and 5th cycles, the lift off area is shifted to lower trough areas. This shift is more in the presence of ECN. Further, the compressibility modulus also shows that in the presence of ECN, the trough area where collapse occurs (indicated by a sharp increase in compressibility modulus) is shifted to lower trough areas (Figure S1).

To quantify the material loss, we calculated the integral area for each surface pressure vs. area compression/expansion cycle, which we have defined simply as “area under the curve”. The log-transformed area under the curve for DPPC:POPG has been shown in Figure 1C. In the case of the first cycle for control, the area under the curve is around 3.45 in the logarithmic scale. After the first compression/expansion cycle, there is a notable drop in the area under the curve. This drop in the value indicates loss in material from the interface. For the second isotherm, the value is about 3.18, and the area keeps dropping till the fifth isotherm, where the value is as low as 2.8. With the addition of the ECNs, we see an increase in the area to about 3.5 for the first isotherm. This suggests the incorporation of the nanoparticles at the interface. The subsequent cycles show decrease in the area with the fifth cycle reaching about 2.75, which is lower than that of the control. 

Therefore, finding the total variance and subsequently taking the square root of the total variance, we obtained the standard deviation of the difference for each cycle. This standard deviation of the difference is mathematically calculated using Equation (2):

(2) $$\sigma_{\mathrm{total}}=\sqrt{(}\sigma_{\mathrm{control}}^{2}+{\;\sigma }_{\mathrm{ECN})}^{2}$$

where, σ_control_ is the standard deviation of the control samples, σ_ECN_ is the standard deviation of the samples, which contain ECN, and σ_total_ is the overall standard deviation of the difference.

### 2.2. Isotherms and Area under the Curve of DPPC:DPPG

**Figure 2:**
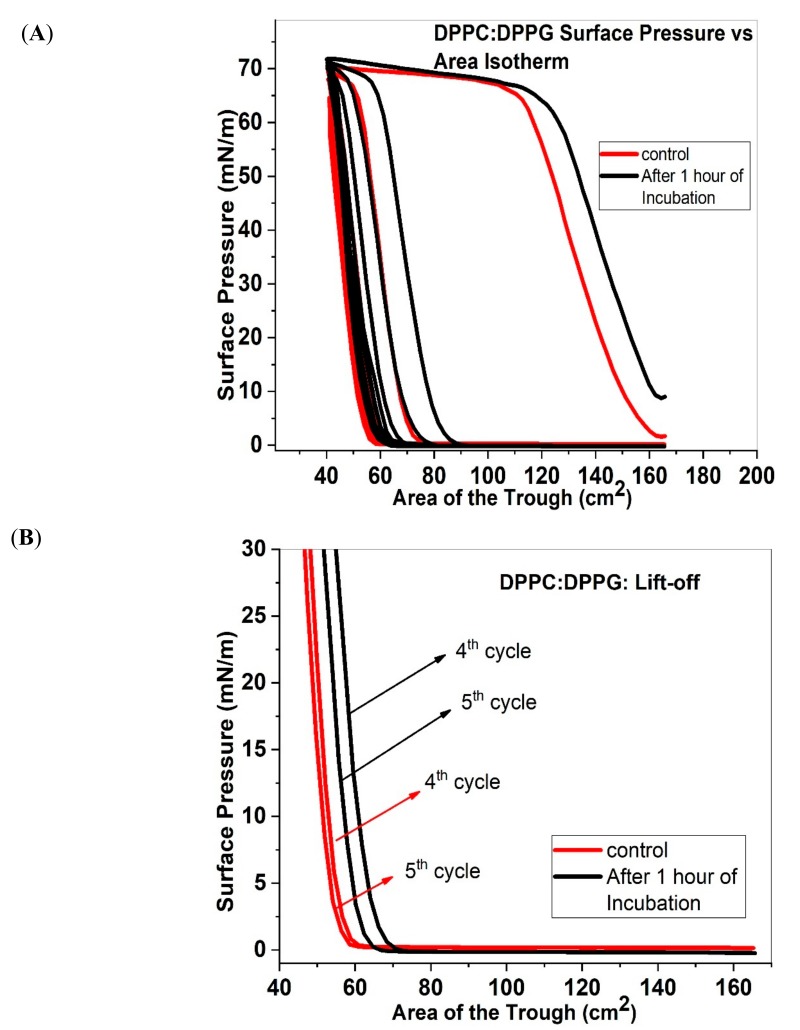

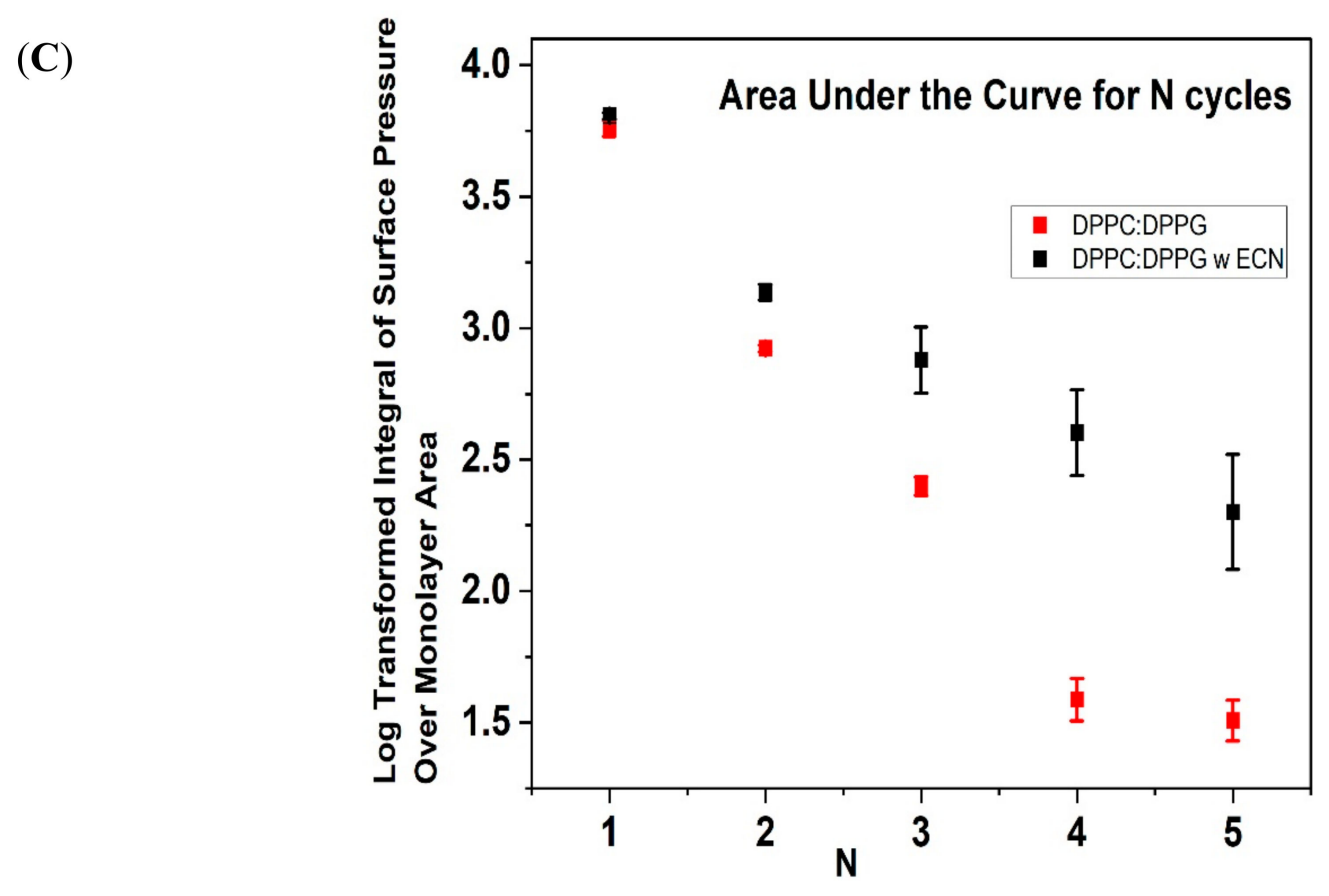
(**A**) Surface Pressure versus area isotherms for DPPC:DPPG (7:3) without (control, red solid line) and with 1 wt% ECN (black solid line). Here again, the samples were compressed and expanded five times to understand loss of material from the surface. An average of three samples was taken to graph the data. (**B**) The initial part of the 4th and 5th compression cycles of DPPC:DPPG monolayers with (black lines) and without (red lines) the ECN, showing the lift off area for each compression cycle. (**C**) Area under the curve as a function of compression/expansion cycle for DPPC:DPPG (7:3) without (control, red square) and with 1 wt.% ECN (black square). In this case, the log-transformed mean and standard deviation of three samples has been shown in the figure.

Figure 2A shows the Π–A isotherms of DPPC:DPPG (7:3) with and without 1 wt% ECN added to the samples. Because the monolayer here comprises of two disaturated phospholipids, the surface gets well packed almost immediately after compression. Unlike the DPPC:POPG monolayer shown above, the LE phase is now short lived and there is no clear LE-LC coexistence phase. Rather, a sharp rise in the surface pressure with compression is seen even at high trough areas, which is typical of this saturated lipid mixture. The surface pressure reaches around 70 mN/m, after which the sample undergoes collapse, with a plateau appearing in the isotherm. Once the sample is expanded, the surface pressure drops to zero with almost very little change in the area of the trough. Beyond the first cycle, DPPC:DPPG lipid mixtures show a significant shift in the compression curve for the second, third and fourth cycle. However, when ECN is added to the samples, there is a shift in the compression curve to higher area of trough for all compression cycles, which is the opposite direction from the previous lipid mixture. However, the shape of the curve is not altered in the presence of ECN, i.e., the phase transformations remain the same as that of the control. Figure 2B further shows that addition of ECN causes the lift-off area to be shifted to higher trough areas for both the 4th and 5th compression cycles, suggesting an increase in the material present at the interface during consecutive cycles when compared with the control system. This trend is also reflected in the compressibility modulus data (see Figure S2), further confirming that the trough area where collapse occurs is also shifted to higher trough areas. 

Figure 2C shows the area under the curve for the two systems studied for multiple compression/expansion cycles. The log-transformed, integral area for the first compression/ expansion cycle of the control lipid mixture shows a higher value of about 3.75 when compared with the DPPC:POPG mixture. However, subsequent compression/expansion cycles demonstrated a steady decrease in area under the curve. For the control sample, by the fifth compression/expansion cycle, the area under the curve is almost zero. Addition of ECN shows an increase in the area of the first cycle compared to the control. The log-transformed, integral area reaches a value of about 3.8. Moreover, while there is a loss in the total area with subsequent cycles, this decrease is more gradual for the lipid mixtures exposed to ECN. Figure 2C shows that the log-transformed area under the curve decreases to a value of 2.5 for DPPC:DPPG films containing ECN, instead of nearly zero, as in case of the control. 

### 2.3. Isotherms and area under the curve of DPPC:POPC

**Figure 3:**
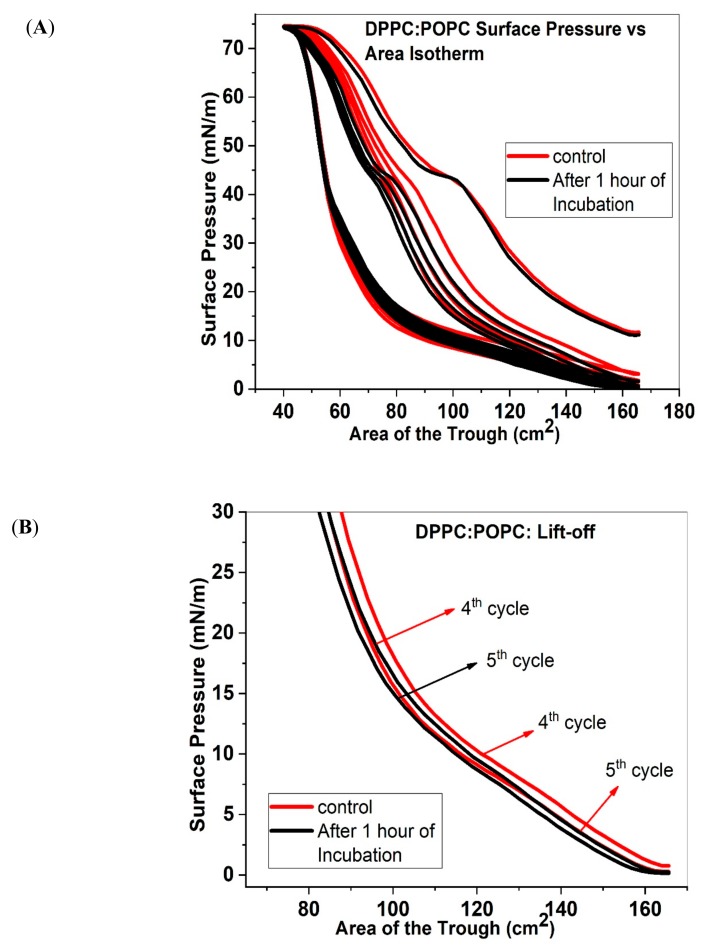

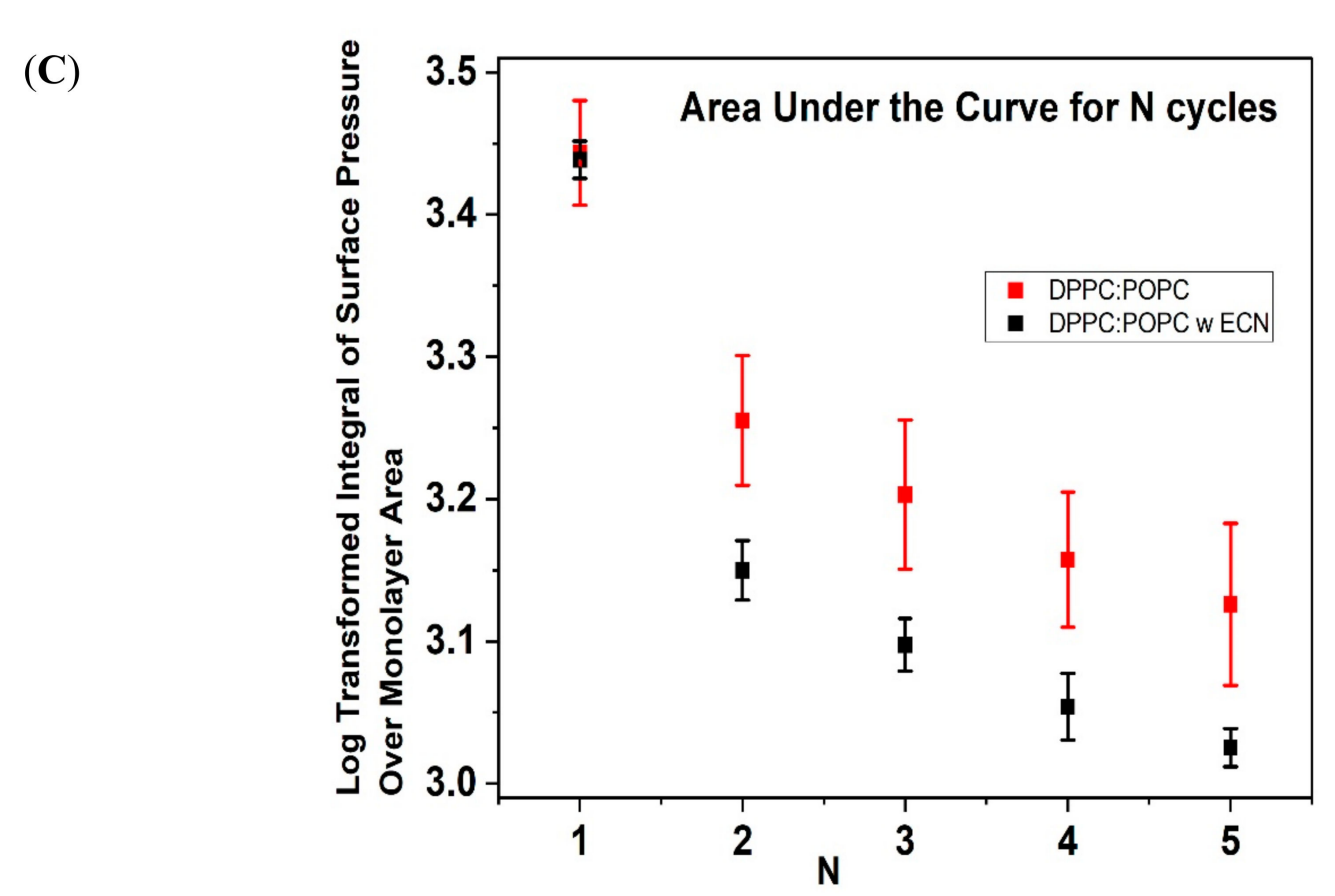
(**A**) Surface Pressure versus area isotherms for DPPC:POPC (7:3) without (control, red solid line) and with 1 wt% ECN (black solid line). Similar to the DPPC:POPG, DPPC:POPC samples were compressed and expanded five times. The data has been represented as the mean of 3 samples. (**B**) The initial part of the 4th and 5th compression cycles of DPPC:POPC monolayers with (black lines) and without (red lines) the ECN, showing the lift off area for each compression cycle. (**C**) Area under the curve as a function of compression/expansion cycle for DPPC:POPC (7:3) without (control, red square) and with 1 wt.% ECN (black square). Three samples were taken to obtain the log-transformed mean and standard deviation.

Figure 3A shows the surface pressure vs. area isotherms for DPPC:POPC films with and without the ECN. In the case of DPPC:POPC, the compression cycles demonstrate the appearance of a distinct plateau around 45 mN/m. This plateau corresponds to the collapse pressure of POPC, thus indicating a rejection of material from the surface into the subphase. However, soon after, the refined isotherm follows a steeper slope reaching surface pressure of above 70 mN/m. Once the monolayer is expanded the surface pressure starts to plummet until it reaches values of 13 mN/m. Interestingly, beyond this initially drop, the slope becomes more gradual. This difference in the compression-expansion curve (hysteresis) appears to be typical for all the different lipid mixtures studied here. It is also interesting to note that initially, addition of ECN shows no change in the compression curve. However, with consecutive compression-expansion cycles, there is a shift of the isotherms to lower areas. A large shift in the isotherms with the addition of ECN, indicates loss in material. Figure 3B also shows the lift off areas for the 4th and the 5th cycles which are found to shift to slightly lower values with the addition of ECNs. Similarly, the compressibility modulus data (Figure S3) shows that the trough area where collapse occurs is shifted to lower values. Figure 1C shows the log-transformed area under the curve for 5 compression/expansion cycles, for lipid mixtures with or without (control) ECN.

The area under the curve for the first cycle of DPPC:POPC control is around 3.45. We again see a large drop in the area as the isotherm goes through a second cycle of compression and expansion. Finally, after the fifth cycle, the value drops to about 3.13. When ECN is added, the first isotherm has an area very similar to that of the control. However, we see a greater reduction in the area as we go through the remaining cycles of compression/expansion. After the fifth cycle, the log-transformed value of the area reduces to 3.03. 

### 2.4. Isotherms and Area under the Curve of DPPC:DPPE

**Figure 4:**
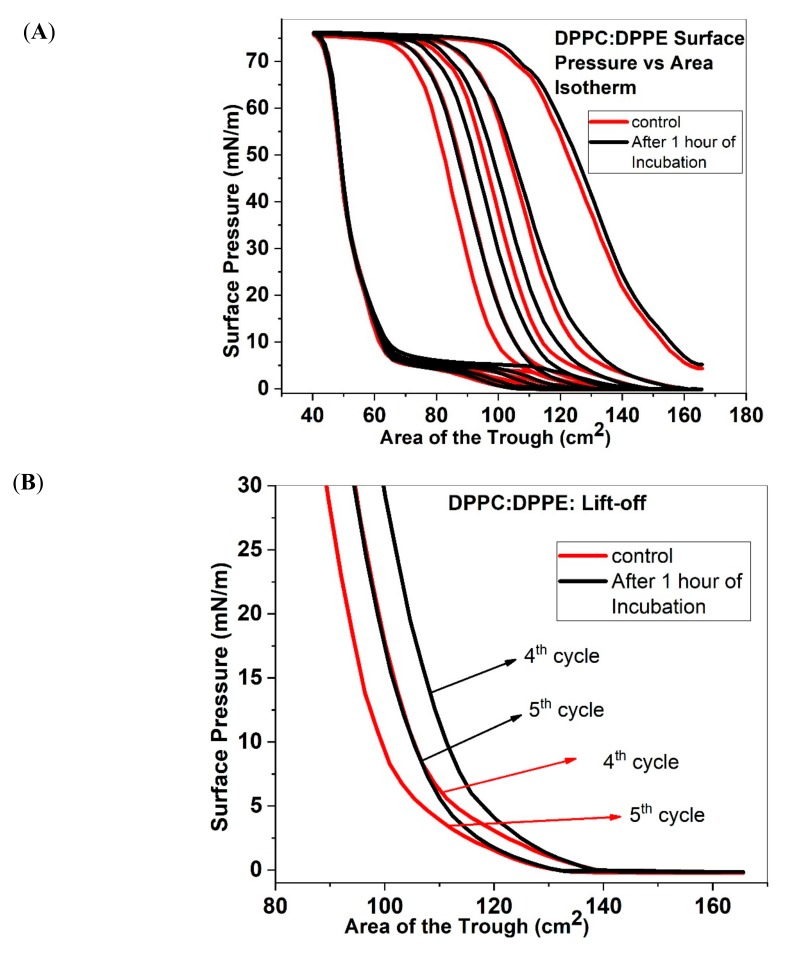

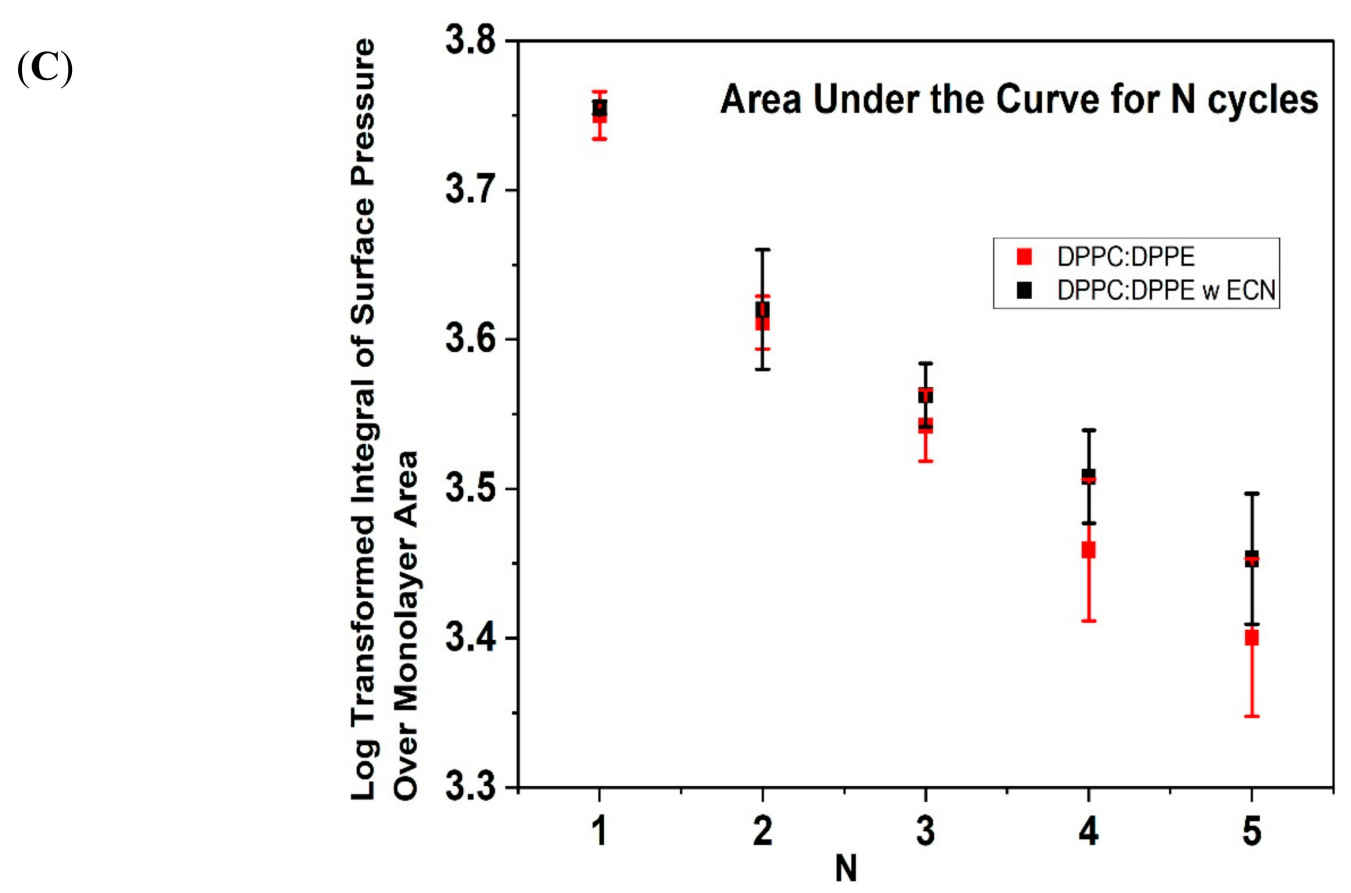
(**A**) Surface Pressure versus area of the trough isotherms for DPPC:DPPE (7:3) without (control, red solid line) and with 1 wt% ECN (black solid line). All samples were compressed and expanded five times. The data has been represented as the mean of 3 samples. (**B**) The initial part of the 4th and 5th compression cycles of DPPC:DPPE monolayers with (black lines) and without (red lines) the ECN, showing the lift off area for each compression cycle. (**C**) Area under the curve as a function of compression/expansion cycle for DPPC:DPPE (7:3) without (control, red square) and with 1 wt.% ECN (black square). The log-transformed mean and standard deviation of three samples have been shown in the figure.

Figure 4 shows the Π–A isotherms of DPPC:DPPE (7:3) with and without 1% ECN added to the sample. The surface pressure rises with a steep slope, rapidly reaching high values, and showing a lack of LE-LC coexistence seen in DPPC:POPG and DPPC:POPC films. Upon reaching the maximum pressure, the isotherm goes through a plateau as the sample collapses. While expanding the monolayer, the surface pressure drops sharply till it reaches around 7 mN/m, and then the isotherm goes through another plateau to reach near zero values. The subsequent cycles move to lower areas of the trough. Addition of ECN causes a shift in the isotherm to higher trough areas. It is important to note that for this shift towards larger areas becomes more pronounced with each subsequent cycle. To better analyze this data, in Figure 4B, we plot the initial section of the surface pressure vs. area curve for the 4th and 5th cycle, which also shows the “lift-off” area. The “lift off area” is shifted to higher values, in the presence of ECN in the lipid films. Similarly, our compressibility modulus data (Figure S4) shows that the trough area where monolayer collapse occurs is shifted to higher values. A further analysis of the area under the curve, presented in Figure 4C, provides a better understanding of the loss. Figure 4C shows the area under the curve for DPPC:DPPE films with and without ECN. For the first compression/expansion cycle of the control, the area reaches about 3.75. While there is a continual loss of material with the subsequent cycles, for the fifth cycle, the area reduces to a value of 3.39, which suggests that this change is not as drastic as the changes seen in DPPC:DPPG films. With the addition of ECN, the first and second cycles have similar area under the curve as that of the control. However, with consecutive cycles, the area under the curve increases with addition of ECN, suggesting more material retention or more packed films at the interface. 

Next, we obtained the differences in the area under the curve between the control and the samples containing ECN for each cycle such that we can have a better understanding of the deviation from the control once ECN is added. 

### 2.5. Difference in the Area under the Curve for the Samples Tested

**Figure 5:**
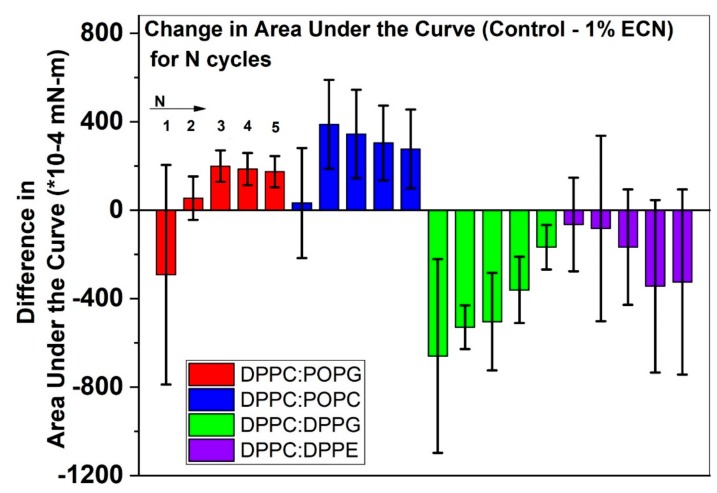
Difference in the area under the curve between control and samples containing 1% ECN for each compression/expansion isotherm. DPPC:POPG is shown by the red bars, DPPC:POPC by blue, DPPC:DPPG by green and DPPC:DPPE by purple. Each bar represents the mean of 3 samples. The control data is independent from that of the samples containing ECN.

The difference in the integral area under the curve between the control and 1% ECN, shown in Figure 5, provides us with the impact of ECN on material loss. Positive values for the difference suggest the presence of less material at the interface when ECN is added, whereas, negative values imply more material is present when ECN is added to the sample. Thus, negative values for this graph is indicative of an improvement in the sample in terms of material retention. In the case of DPPC:POPG, we see mostly positive changes when ECN is added. Only for the first cycle, we have a mean value of –0.03 mN-m. In the case of DPPC:POPC, we see positive values for this difference for all the cycles. However, when ECN is added to the disaturated material, we start observing negative values for each cycle. In the case of DPPC:DPPG, we see negative values for all the cycles. In the case of DPPC:DPPE also, the mean for all the cycles has negative values. Therefore, ECN shows a beneficial impact on these lipid mixtures containing only saturated lipids (which possibly lead to more well-packed films). 

### 2.6. Effective Molecular Area for DPPC:POPG and DPPC:POPC

**Figure 6:**
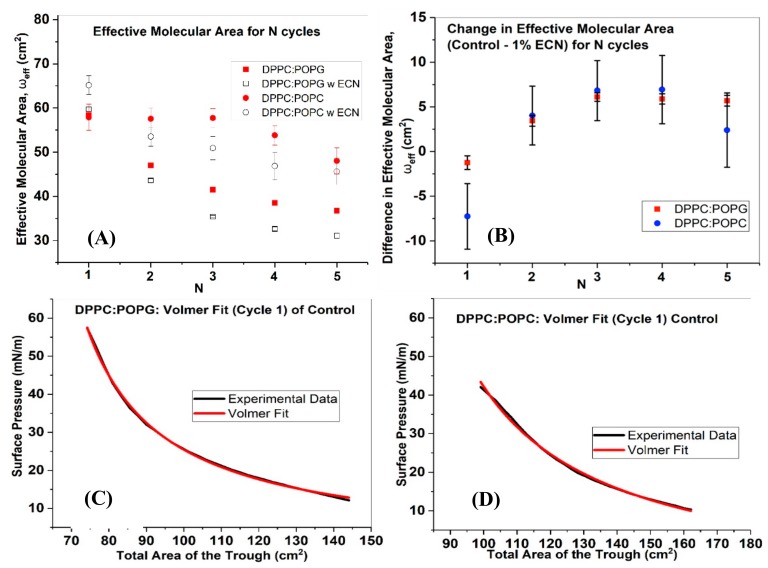
Effective molecular area and difference in effective molecular area derived by fitting Volmer’s equation to Π–A isotherms. (**A**) One of the parameters of equation 3 is the effective molecular area, denoted by ω_eff_. ω_eff_ as a function of compression/expansion cycle was plotted for DPPC:POPG (7:3) control (closed square), DPPC:POPG (7:3) with 1% ECN (open square), DPPC:POPC (7:3) control (closed circle) and DPPC:POPC (7:3) with 1% ECN (open circle). (**B**) A difference in ω_eff_ between the control and1% ECN has been plotted for DPPC:POPG (7:3) (red square) and DPPC:POPC (blue circle). The data has been represented as the mean and standard deviation of 3 samples. (**C**) and (**D**) Experimental data and Volmer fits to the first cycle for DPPC:POPG and DPPC:POPC demonstrate the quality of the fit.

For lipid monolayers with a well-defined LE phase and/or LE-LC coexistence, material loss can be also be characterized by the parameter ω_eff_. A decrease in *ω_eff_* with each subsequent cycle of compression/expansion indicates a loss in material. In the case of DPPC:POPG control, the area drops from around 58 cm^2^ to 37 cm^2^ over five compression/expansion cycles. With the addition of ECN to the sample, the effective molecular area drops to as low as 33 cm^2^ (lower than the control system). In the case of DPPC:POPC control, the loss is smaller than that for DPPC:POPG control. From around 57 cm^2^ initially, ω_eff_ drops to about 48 cm^2^ over five compression/expansion cycles. With the addition of ECN to DPPC:POPC the ω_eff_ for the first cycle is higher than that of the control. However, from 65 cm^2^ at the first cycle, the ω_eff_ drops to about 47 cm^2^ after the 5th cycle. Figure 6B shows the change in effective molecular area between the control and 1% ECN for each cycle for DPPC:POPG and DPPC:POPC. A difference between the effective molecular areas for the control and the samples with 1% ECN was obtained for each cycle. A larger deviation from the control suggests loss in material. Initially, more effective area is available for DPPC:POPC with 1% ECN than it’s DPPC:POPG counterpart. However, from the 2nd cycle onwards, the value drops drastically for DPPC:POPC with 1% ECN. Therefore, the data suggests that ECN has an immediate impact on DPPC:POPC. At this point, it should be mentioned that since the DPPC:DPPG and DPPC:DPPE films are already beyond the LC phase, we did not model their isotherms using the Volmer’s equation.

### 2.7. Fluorescence Micrographs of the Samples Tested

**Figure 7:**
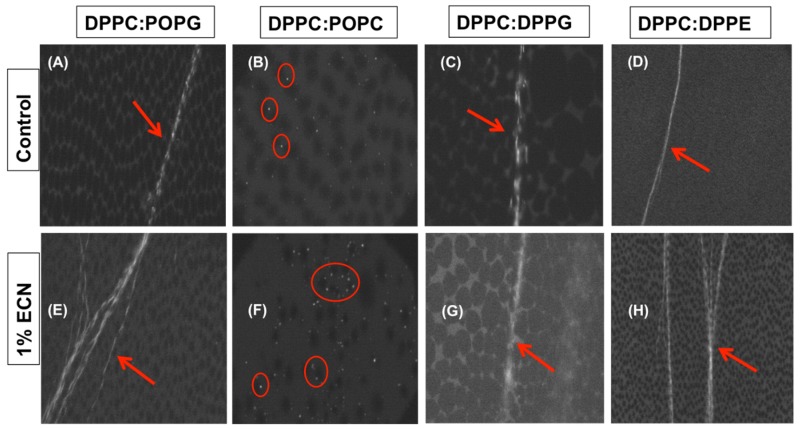
Fluorescence micrographs taken after monolayer collapse (**A**) DPPC:POPG (7:3) control, (**B**) DPPC:POPC (7:3) control, (**C**) DPPC:DPPG (7:3) control, (**D**) DPPC:DPPE (7:3) control, (**E**) DPPC:POPG (7:3) with 1 wt% ECN, (**F**) DPPC:POPC (7:3) with 1% ECN, (**G**) DPPC:DPPG (7:3) with 1 wt% ECN and (**H**) DPPC:DPPE (7:3) with 1% ECN. The arrows point out folded, reversible, collapse features on the monolayer. The circles highlight the vesicles formed in the monolayer after collapsing.

Figure 7A,E display the monolayer collapse for DPPC:POPG control and with 1% ECN. In the case of DPPC:POPG control Figure 7A, the monolayer collapses with the formation of folded features, which have been pointed out by the arrow. These folded structures can be easily spotted in an otherwise dark monolayer. While most of the monolayer is extremely packed, the regions where the collapse features exist appear as bright streaks, perpendicular to the direction of compression, spanning across the width of the micrograph. Figure 7E suggests that these features remain intact when ECN is added to our sample. The rest of the image appears dark because the monolayer becomes extremely packed once the surface pressure reaches high values. Similar to the DPPC:POPG monolayer, the DPPC:DPPG control (Figure 7C) collapses with the formation of reversible collapse features. Moreover, addition of ECN (Figure 7G) to DPPC:DPPG doesn’t alter the collapse mechanism in this case. Furthermore, Figure 7D shows the collapse features for DPPC:DPPE control. Here too we observe reversible collapse features, and when ECN is added (Figure 7H), the reversible collapse features are still present. Figure 7B,F on the other hand, shows the collapse features in the case of DPPC:POPC control and with 1% ECN. The mechanism of collapse is different in the case of DPPC:POPC. Here too we find the monolayer to be packed, and therefore, the majority of the monolayer appears dark. However, instead of the collapse features that was observed in the case of DPPC:POPG, bright specks appear in the monolayer. These specks in the monolayer of DPPC:POPC control (Figure 7B), have been highlighted by circles and are believed to be vesicles. When ECN is added to the sample, the specks appear to increase in number. However, the overall mechanism of collapse remains the same for all the samples when 1% ECN is added. 

## 3. Discussion

In this section we discuss the major implications of our results presented above. In the present work, the impact of nanoparticle-phospholipid interactions on multiple compression/expansion cycles were studied, along with direct visualization of the phospholipid monolayer morphology at monolayer collapse, to understand the impact of nanoparticles on different mechanisms of monolayer collapse. Further, we also explored how variations in monolayer composition demonstrate differences in the response to nanoparticle interactions. Multiple compression/expansion cycles are relevant for lung surfactants because a loss in the material is encountered after each cycle of breathing. A greater loss in the material with the addition of nanomaterial suggests a detrimental impact of the particles on the proper functioning of the surfactants. Kodama et al., used multiple compression/expansion cycles to discuss how particle size, ranging from 20 nm to 1.0 μm, affect the phase behavior of surfactant monolayers [30]. While several previous studies have focused on the effect of nanomaterial on the surface activity and surface morphology of native as well as model lung surfactant monolayers [13,16,31], to the best of our knowledge, the impact of nanoparticles on collapse mechanisms in lipid systems containing anionic, zwitterionic or mixed anionic/zwitterionic lipids is currently unknown.

Therefore, in this work, we use multiple compression/expansion cycles to understand how the same nanoparticle influences the mechanisms of collapse in four different lipid systems that demonstrate different collapse mechanisms due to differences in headgroup charge and tail saturations. Surface pressure vs. area isotherms were used to calculate the total area under the curve as well as a shift in the lift-off area in compression isotherms. An increase in the total area under the curve indicates an increase in material retention at the air-water interface, while a shift in the lift-off area to higher areas suggest more material at the interface. Additionally, fluorescence images provide direct visual evidence of the mechanisms of collapse (formation of collapse cracks vs. vesicles).

Overall, our data suggest that the impact of ECN on the reversibility of phospholipid monolayers is dependent on both the charge of the monolayer as well as the lipid tail saturation. Fluorescence imaging provides clear evidence that addition of small amounts of these negatively charged nanoparticles did not change the actual mechanism of collapse for all the different systems studied. However, direct visual imaging fails to reveal the subtle changes observed when ECN interacts with phospholipid mixtures. These ECN induced subtle changes in monolayer collapse could only be captured by analyzing multiple cycles of compressions and expansions.

### 3.1. Membrane Packing Influences ECN’s Ability to Modulate Monolayer Collapse in Mixed Lipid Systems

We start by analyzing our results for the DDPC:POPG and DPPC:DPPG mixtures. Both of these mixtures are often used as model LS mixtures and are therefore biologically relevant. It is important to note that while both lipid systems have an overall negative charge due to the PG headgroup, the difference lies in the tail saturation. DPPC:DPPG lipid mixture is more packed than DPPC:POPG, due to the unsaturation in the POPG tails. A comparison of our fluorescence images for these two systems with and without the nanoparticles show that both these mixtures undergo reversible monolayer collapse. However, an analysis of the multiple compression/expansion studies show that when nanoparticles are added to lipid mixtures, an increase in material retention is measured between the compression/expansion cycles containing saturated lipids only. On the other hand, for the mixtures containing unsaturated POPG lipids, a loss in material retention is measured. Further, for the saturated DPPC/DPPG lipid mixture, we find that the trough area where “lift off” occurs is shifted to higher areas, while the “lift off” area is shifted to lower areas for the mixtures containing unsaturated POPG. Similarly, our compressibility data shows that the trough area where monolayer collapse occurs is also shifted to higher area for lipid films containing only saturated lipids, while films containing a mixture of saturated and unsaturated lipids have to be compressed further before they reach collapse, when ECN is added to them suggesting there is less reincorporation of material than the control system. These results together suggest that saturated lipids better enable incorporation of the nanoparticles into the monolayers, and thus have a positive impact on the overall performance. On the other hand, the presence of unsaturated POPG lipids seem to cause a detrimental effect on the monolayers’ ability to collapse reversibly and retain material at the interface. Based on this comparison we conclude that interactions of the nanoparticles with unsaturated lipid mixtures cause increased material loss during monolayer collapse.

This conclusion is further validated by our results for the DPPC:POPC system. Addition of ECN to this monolayer system shows the highest loss of material among all the systems studied here. One possible explanation for this behavior is the possible changes to the line energy of the lipid monolayer induced by the nanoparticles due to their interactions with the lipid headgroups. We have previously shown that in case of lipid monolayers containing POPG, the positively charged nanoparticles avoid the negatively charged POPG lipids that are present in the more fluid liquid-expanded (LE) region and cause a lowering of the line tension. Similarly, for the DPPC:POPC system, we have previously shown that ECNs induce a lowering of the line tension between domain boundaries in this system, as evidenced by a transition in the domain morphology from the signature kidney-bean domains seen in DPPC systems, to domains with arms. On the other hand, in case of the saturated DPPC:DPPG lipid system, addition of ECN caused an increase in the line energy of the system by partitioning into the more fluid LE phase. These results together suggest that ECN-induced lowering of the line tension of monolayers containing mismatched lipid tails adversely impact their ability to collapse reversibly.

### 3.2. Lipid Headgroup Charge Influences the Ability of ECN to Alter Monolayer Collapse

To explore if ECN induced changes in monolayer collapse depends on the presence of anionic lipid headgroups, we compare our results for lipids with zwitterionic vs. anionic lipids, both for saturated and unsaturated lipid systems.

A comparison between the DPPC:POPG and DPPC:POPC systems show that addition of ECN induces a higher loss in material between multiple cycles for the zwitterionic DPPC:POPC system, when compared with DPPC:POPG monolayers containing a net negative charge. Since both of these systems have the same combination of tail saturation, we can attribute the difference in ECN’s influence on monolayer collapse to a difference in the lipid headgroup.

The negative charge on the ECN causes it to avoid the PG headgroup and therefore interact less in the monolayer. This lack of interaction may cause less loss of material between multiple compression/expansion cycles. A comparison of DPPC:DPPG and DPPC:DPPE monolayers show that the increase in material retention induced by ECNs is also more pronounced in the lipid monolayer containing anionic headgroup (note that there is no mismatch in the lipid tails in these two systems). Together, these results suggest that negatively charged nanoparticles produce a stronger change in monolayer collapse for lipid monolayers containing anionic lipids.

### 3.3. Fluorescence Imaging of the Monolayer Shows that the Mechanism of Monolayer Collapse is not Altered by ECN

Gopal et al. have shown that DPPC:POPG monolayers, laterally compressed under conditions, which are similar to our study, collapses via reversible folding mechanism [32]. These reversible folds appear as bright streaks perpendicular to the direction of compression, and range from 100 μm to about 1 mm in length. These reversible structures unfold when the monolayer is expanded, and material reincorporates into the monolayer without notably altering the morphology. Our study shows the occurrence of similar collapse features in the case of DPPC:POPG, DPPC:DPPG and DPPC:DPPE. With the addition of ECN, bright streaks were again seen spanning across the fluorescence micrograph, suggesting that the monolayer retains this collapse feature in all three lipid systems. To prove that this behavior can be reproduced regardless of the mechanism of collapse, we imaged DPPC:POPC monolayers in the absence and presence of ECNs. DPPC:POPC monolayers collapses with the formation of vesicles, which appears as bright specks on the monolayer. These bright specks are thought to be globular vesicles that usually detach from the monolayer [32]. Large vesicles usually end up detaching from the monolayer making the collapse irreversible. In the case of DPPC:POPC control, the formation of the vesicles suggests irreversibility of the monolayer which was also confirmed by the loss of area using compression/expansion isotherms. When ECN was added to the DPPC:POPC mixture, more vesicles appeared on the surface at monolayer collapse. This confirms an increase in the loss of material from the surface when they are compressed beyond the collapse pressure. Again, this loss of material confirms what we inferred, based on our analysis of the isotherm data. Therefore, through fluorescence images, we can conclude that ECN doesn’t alter the mechanism of collapse regardless of the phospholipid it encounters. However, thorough analysis of the compression/expansion cycles over many cycles presents subtle differences in ECN–induced changes to monolayer collapse.

## 4. Materials and Methods

### 4.1. Materials

The phospholipids, 1,2-dipalmitoyl-sn-glycero-3-phosphocholine (DPPC), 1-palmitoyl-2-oleoyl-sn-glycero-3-phospho-(1’-rac-glycerol) (sodium salt) (POPG), 1-palmitoyl-2-oleoyl-glycero-3-phosphocholine (POPC), 1,2-dipalmitoyl-sn-glycero-3-phospho-(1′-rac-glycerol) (sodium salt) (DPPG), and 1,2-dipalmitoyl-sn-glycero-3-phosphoethanolamine [DPPE] were obtained from Avanti Polar Lipids (Alabaster, AL, USA). The phospholipid mixtures were purchased in chloroform mixtures at concentrations of 5 or 25 mg/mL. The phospholipid dye that was used in our study, Texas red 1,2-dihexadecanoyl-sn-glycero-3-phosphoethanolamine, triethylammonium salt (TXR-DHPE), was obtained from Life Technologies (Invitrogen, Grand Island, NY, USA). The engineered carbon nanodiamonds were procured from Microdiamant (Lengwil, Switzerland). Details about the physical properties of these ECNs have been published before. Briefly, the size of the ECN is 240 nm in the organic mixture, and the accompanying polydispersity is 0.35. Furthermore, the ECN used in this study is negatively charged with a zeta potential of −28 mV.

The phospholipids as well as the dye were diluted to 1 mg/mL in high-performance liquid chromatography (HPLC)-grade chloroform before using them in our studies. ECN suspensions were also prepared in the chloroform:methanol solutions. Chloroform, methanol, acetone, and isopropanol used in this study for preparing samples and cleaning equipment, were purchased from Thermo Fisher Scientific Inc. (Pittsburgh, PA, USA). The water, used as the cleaning agent and sub-phase, had a resistivity of 18.2 MΩ/cm), which was prepared in a Millipore gradient system (Billerica, MA, USA).

### 4.2. Methods

#### 4.2.1. Sample Preparation

Table 1 shows the phospholipid-ECN solutions used in our study. Solutions of DPPC:POPG, DPPC:POPC, DPPG mixtures (7:3 by weight) were prepared in chloroform. 1 weight % TXR-DHPE dye (dissolved in 4:1 chloroform:methanol mixture) was added to the lipid samples. Carbon nanodiamonds suspensions were sonicated for 2 h, and immediately afterward, stoichiometric volumes were added to the lipid mixtures for the experiments involved in this study.

#### 4.2.2. Langmuir Studies

Nanodiamonds were mixed with the lipid samples at a concentration of 1 wt.%. While in a previous study varying concentrations were used, in this work we chose to use 1 wt.% based on our previous work [25]. The ECN were sonicated for 2 h, added to the lipid mixtures and added dropwise on the surface of ultrapure water contained in a Langmuir Ribbon Trough, purchased from Biolin Scientific Inc. (Phoenix, AZ, USA). The trough consists of movable ribbon that can compress/expand such that the molecules on the surface can go through different phases. The multiple compression and expansion cycles serve as a model that mimics the decrease and increase in the alveolar area with exhalation/inhalation. The maximum area of the trough is 166 cm^2^, and the minimum area is 46 cm^2^, which provided the samples with enough area to reach high surface pressure values upon compression. Furthermore, the ribbon is computer controlled to move at a uniform rate with the help of the software supplied by Biolin Scientific Inc. Material was added to the surface in such a way that the starting surface pressure was around 10 mN/m. Although surface pressure vs. area studies are often started at an initial surface pressure of 0 mN/m, in this work a higher starting surface pressure was used to ensure that collapse pressure was reached for all five compression cycles (see Figure S5). After spreading the sample solution on the water surface and before starting the compression/expansion cycles, the chloroform was allowed to evaporate for 20 min. This waiting period also provides sufficient time for the monolayer to spread uniformly on the surface. After the 20-min period, the ribbon was moved at the rate of 125 mm/min for the compression/expansion isotherms. However, in the case of fluorescence imaging, a slower rate of 7.0 mm/min was used for clarity.

#### 4.2.3. Fluorescence Imaging

The trough is also coupled with an Eclipse fluorescence microscope (Nikon, Japan) for visualizing the surface morphology of the monolayer. The microscope is equipped with a 40×–long working distance objective lens along with motorized-focusing capabilities that allow us to monitor the surface of the monolayer continuously. A dichroic mirror/barrier filter assembly is used in this setup to direct the excitation light perpendicular to the monolayer, whereupon, the emitted light is filtered and captured by the microscope coupled with a fast CCD camera (Andor Luca, Twin Cities, MN, USA). For our purposes, we recorded images in sequences of five to observe the morphology.

### 4.3. Theoretical Analysis

#### 4.3.1. Analysis of Material Loss

##### Area under the Curve and Percentage Recovery

Surface pressure versus area isotherms can provide evidence of interaction between molecules at the interface. With the progression of compression/expansion cycles, the isotherm for most Langmuir monolayers is expected to shift to lower areas, which is indicative of the loss in material once the film is compressed beyond its collapse pressure. Further, it is important to notice that a Langmuir film compressed beyond its collapse pressure shows a difference in its Π-A isotherm between the compression and expansion cycle. This difference in the two Π-A isotherms between the compression and expansion cycle is often referred to as the hysteresis in the surface tension. Almost all lung surfactant mixtures demonstrate this hysteresis [29]. Therefore, we focused on analyzing the effect of addition of nanoparticles to the hysteresis of the different lipid mixtures. Hysteresis corresponds to the area enclosed within the two curves, which forms an envelope. It can be measured from the P-A isotherms, by calculating the polygon area (integral area under the curve) function in Origin 2017. A lowering of such area shows the extent of material loss, which is one of the primary focuses of our study.

#### 4.3.2. Model Based on Volmer’s Equation of State for the Prediction of the Π-A Isotherm at the Region of 2-Dimensional Coexistence Phase

Equations of state [EOS] at the air-water interface can be used to predict the two-dimensional phase coexistence region of the Π-A isotherms of amphiphilic monolayers. Fainerman and Volhardt have described such EOS for insoluble Langmuir monolayers, which is capable of predicting the Π-A isotherms at the gaseous region as well as the 2D phase transition for single amphiphile [33]. Another theoretical model published by the same group describes the liquid expanded region of the Π-A isotherms of different amphiphilic molecules [34]. Recently, Ghazvini et al. used the theoretical model proposed by Feinerman and Volhardt to understand the impact of pH on the packing of phospholipid membranes [35]. Furthermore, these equations are also capable of predicting the material loss. Kodama used a modified form of this equation to calculate the material loss in a lipid mixture due to exposure to nanoparticles of different sizes. Here, we represent the key equations that have been used in the present work to quantify the material loss at the interface.

Mathematically, Volmer’s equation is expressed as follows (Equation (3)):

(3) $$\Pi =\frac{kT\omega }{\omega_{o}}\left(\frac{1}{A-\omega \;}\right)-\Pi_{coh}$$

where π represents surface pressure of the monolayer, k is the Boltzmann constant, T is the temperature, ω is the average effective molecular area of the insoluble species, ω_o_ is the molecular area per water molecule, A is the available surface area per insoluble molecule, and π_coh_ is the cohesion pressure. However, the available surface area, A, requires the knowledge of the number of molecules, n, at the surface. A can be related to the trough area, A_T_, as follows:

A = A_T_/*n* (4)

Using the definition for A from Equation (4), in Equation (3), Kuo et. al. characterized the material loss from the surface [27] using Equation (4) below:

(5) $$\Pi =\frac{kT}{\omega_{o}}\left(\frac{\omega_{eff}}{A_{T}-\omega_{eff}\;}\right)-\Pi_{coh}$$

Here, ω_eff_ is an effective total molecular area, and is given by Equation (6):

ω_eff_ = *n* ω (6)

Equation (5) can be directly fitted to the π-A isotherms. Additionally, ω_eff_ helps us identify the extent of material loss.

However, it should be noted that equation 4 works only in the LE region. Since lipid mixtures with saturated lipids only do not often have a large LE phase at room temperature, we used equation 4 to calculate material loss in lipid mixtures containing unsaturated lipids.

## 5. Conclusions

In conclusion, our results, discussed above, demonstrate that the negatively charged ECN has varying impact on collapse of phospholipid monolayers.

While our fluorescence images after the first compression cycle shows that ECNs do not impact the mechanism of monolayer collapse, a detailed analysis of the surface pressure area (Π-A) isotherms over five consecutive compression/expansion cycles show that lipid mixtures containing ECN show a difference in material loss and material re-incorporation between the different lipid systems. Specifically, we find that when interacting with phospholipid mixtures containing saturated lipids only, ECN causes a net positive effect on monolayer collapse by improving the adsorption of material from the subphase. On the other hand, ECN causes more material loss between cycles for systems containing unsaturated lipids. Further, we find that for both saturated and unsaturated lipid mixtures, the presence of negative charge enables more retention/re-incorporation of material into the monolayer interface, when compared with the neutral lipids. Finally, ECN has the most negative impact on neutral unsaturated DPPC:POPC lipid mixture and an improvement in the compression/expansion isotherms for negative saturated DPPC:DPPG mixture. Since several of these lipid mixtures are used as model lung surfactant mixture, we conclude that it is crucial to study the impact of nanoparticles on multiple compression/expansion cycle to gain knowledge on the toxicity of nanomaterial on lung surfactants. Further, since lung surfactants contain unsaturated anionic lipids, our results suggest that the monolayer collapse mechanism of lung surfactants may not be impacted significantly due to the presence of nanoparticles. However, it is important to note here that one must be careful when drawing conclusions from model systems such as used here, since we have shown that the composition of the lipid mixtures play an important role when interacting with the nanoparticles. Further, the rate of compression may also play a role. The rate of compression used in these experiments does not reflect the dynamic nature of compression cycles that lung surfactants undergo during breathing and may impact the results. Therefore, the impact of nanoparticles on lipid mixtures during quasi-static vs. dynamic compressions should also be considered. In closing, our results clearly establish that when studying interactions between nanoparticles and model lipid mixtures, differences due to the headgroup charge and tail saturation of the lipid mixtures should also be accounted for, before drawing generalized conclusions about nanotoxicity of a material.

## Figures and Tables

**Table 1:** The table lists the samples that have been used in the study. The phospholipid mixtures were taken in the ratio of 7:3 by weight, keeping the percentage of DPPC maximum. The samples with 1 weight % ECN were compared with their counterpart(control)that had no ECN in the mixture.

Phospholipid Composition (Ratio of 7:3 by Weight)	ECN Percentage (wt. %)	
**DPPC:POPG**	0 (Control)	1
DPPC:POPC	0	1
DPPC:DPPG	0	1
DPPC:DPPE	0	1

## Supplementary Materials

The following are available online at https://www.mdpi.com/1420-3049/25/3/714/s1, Figure S1: Compressibility modulus for the fourth and fifth compression cycles for DPPC:POPG films with and without ECN, Figure S2: Compressibility modulus for the fourth and fifth compression cycles for DPPC:DPPG films with and without ECN, Figure S3: Compressibility modulus for the fourth and fifth compression cycles for DPPC:POPC films with and without ECN. Figure S4: Compressibility modulus for the fourth and fifth compression cycles for DPPC:DPPE films with and without ECN. Figure S5: Surface Pressure vs. trough area isotherm for DPPC:POPG films using two different starting surface pressures.
