# Supplementary material for: Impact of Engineered Carbon Nanodiamonds on the Collapse Mechanism of Model Lung Surfactant Monolayers at the Air-Water Interface
Source: Molecules. 2020 Feb 7;25(3):714. doi: 10.3390/molecules25030714 (PMC7037128; doi:10.3390/molecules25030714)
Supplement: Supplementary file 1 [file molecules-25-00714-s001.pdf]

Supplementary Information:

Compressibility Modulus Calculations:

The isothermal surface compressibility modulus of a two-dimensional film is a measure of the films ability to store mechanical energy as stress when it is subjected to dilatational stresses. Thermodynamically, isothermal compressibility modulus *k, is defined as :*

$$k = 1/A(\partial A/\partial \Pi)$$

The isothermal bulk modulus, β , the inverse of compressibility, is mathematically defined as :

$$\beta = 1/A(\partial \Pi/\partial A)$$

can be easily calculated from the surface pressure vs. area isotherms.

It is important to note that since k *and* β are both second order derivatives of the free energy, a dip in the β vs. A profile signifies a first order phase transition. Therefore, this profile can be used to identify monolayer collapse.

In figures S1-S4, we show ECN induced changes to the compressibility modulus profiles for the different lipid systems. These graphs provide further evidence of ECN induced changes to the collapse mechanisms after multiple compression-expansion cycles.

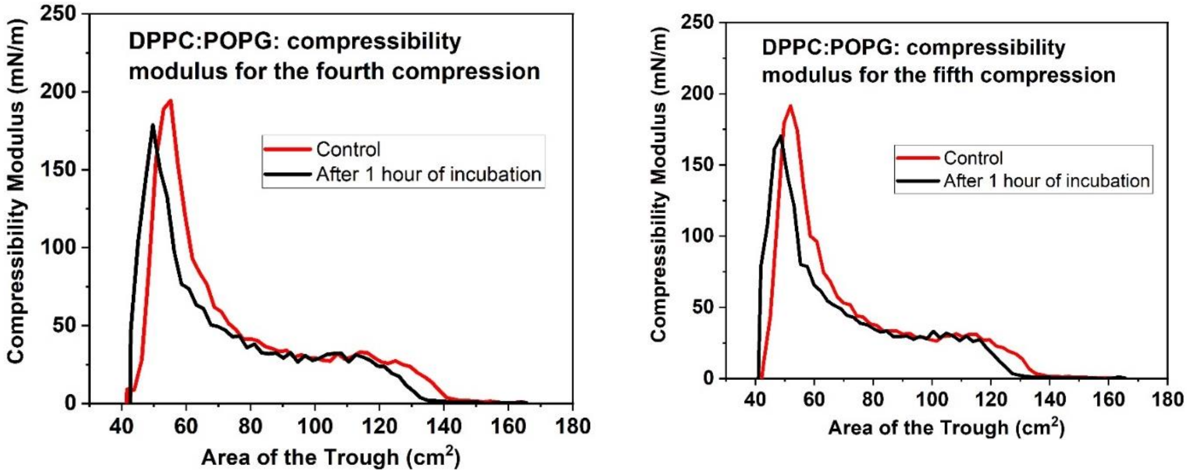

Figure S1: Compressibility modulus for the fourth and fifth compression cycles for DPPC:POPG films with and without ECN.

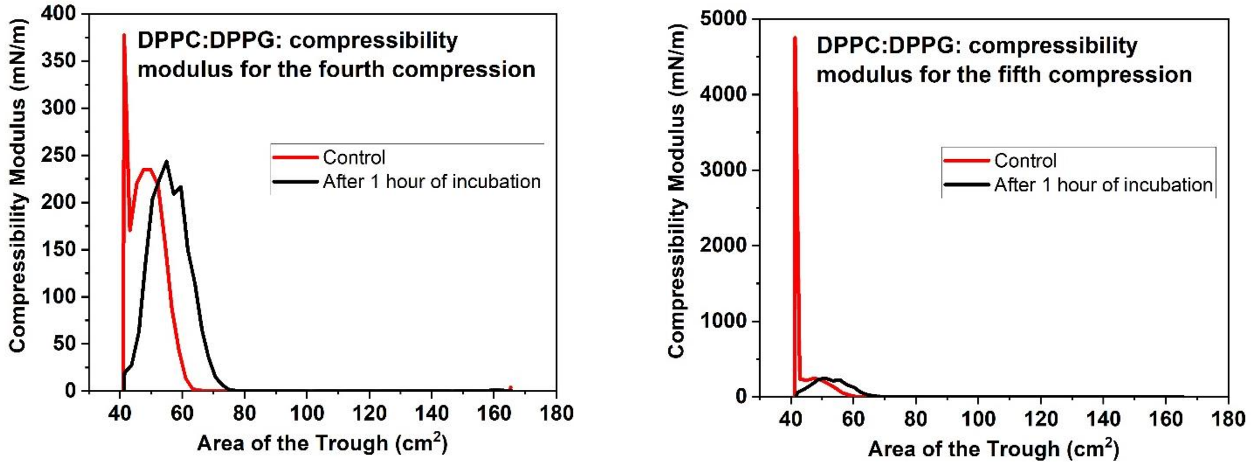

Figure S2: Compressibility modulus for the fourth and fifth compression cycles for DPPC:DPPG films with and without ECN.

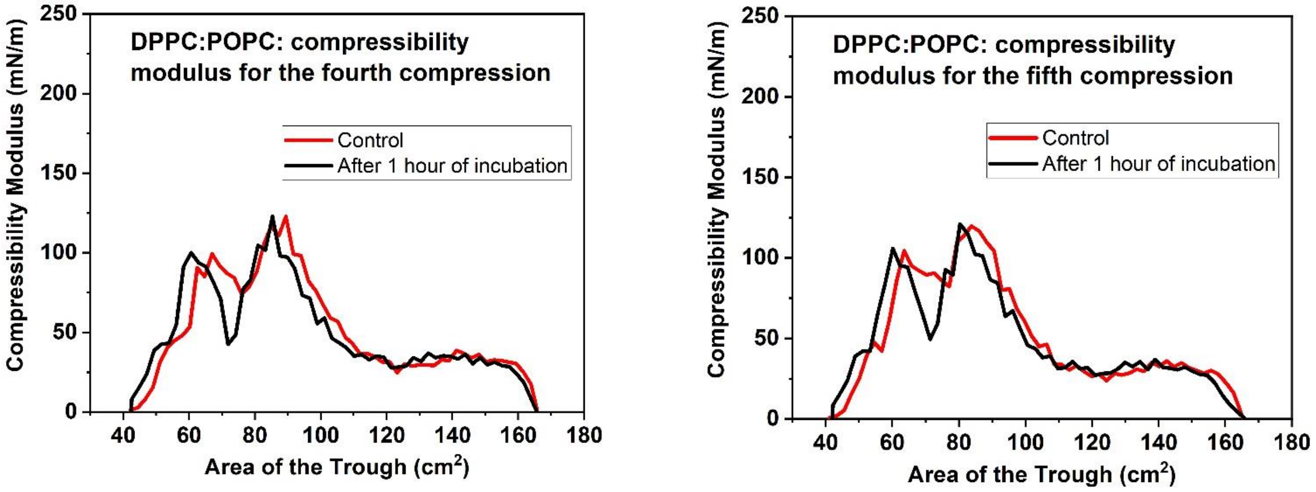

Figure S3: Compressibility modulus for the fourth and fifth compression cycles for DPPC:POPC films with and without ECN.

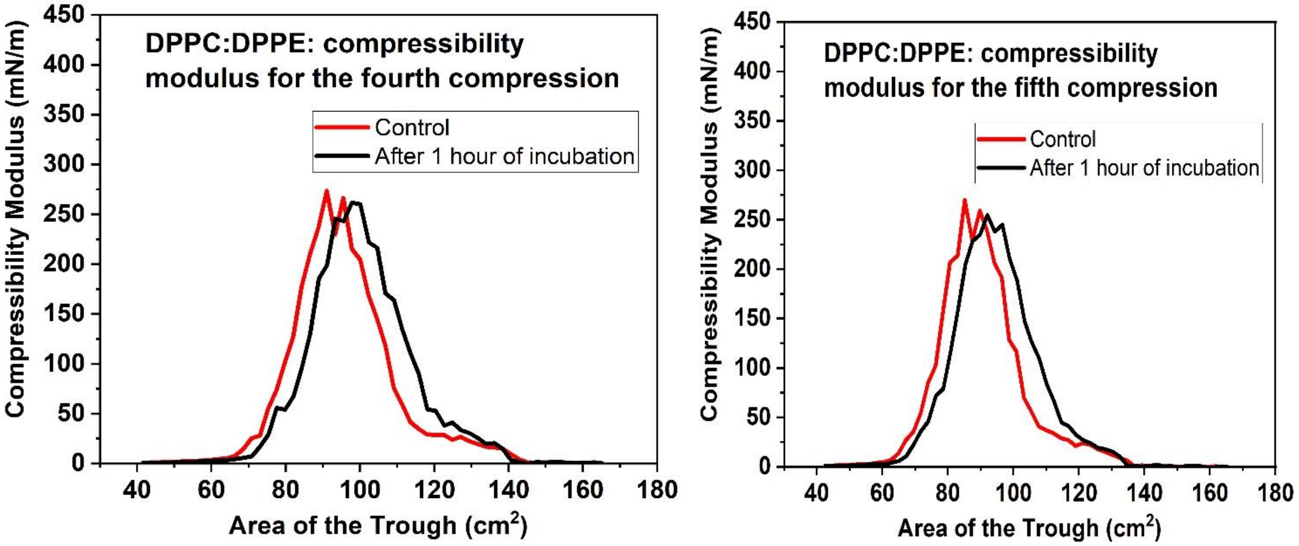

Figure S4: Compressibility modulus for the fourth and fifth compression cycles for DPPC:DPPE films with and without ECN.

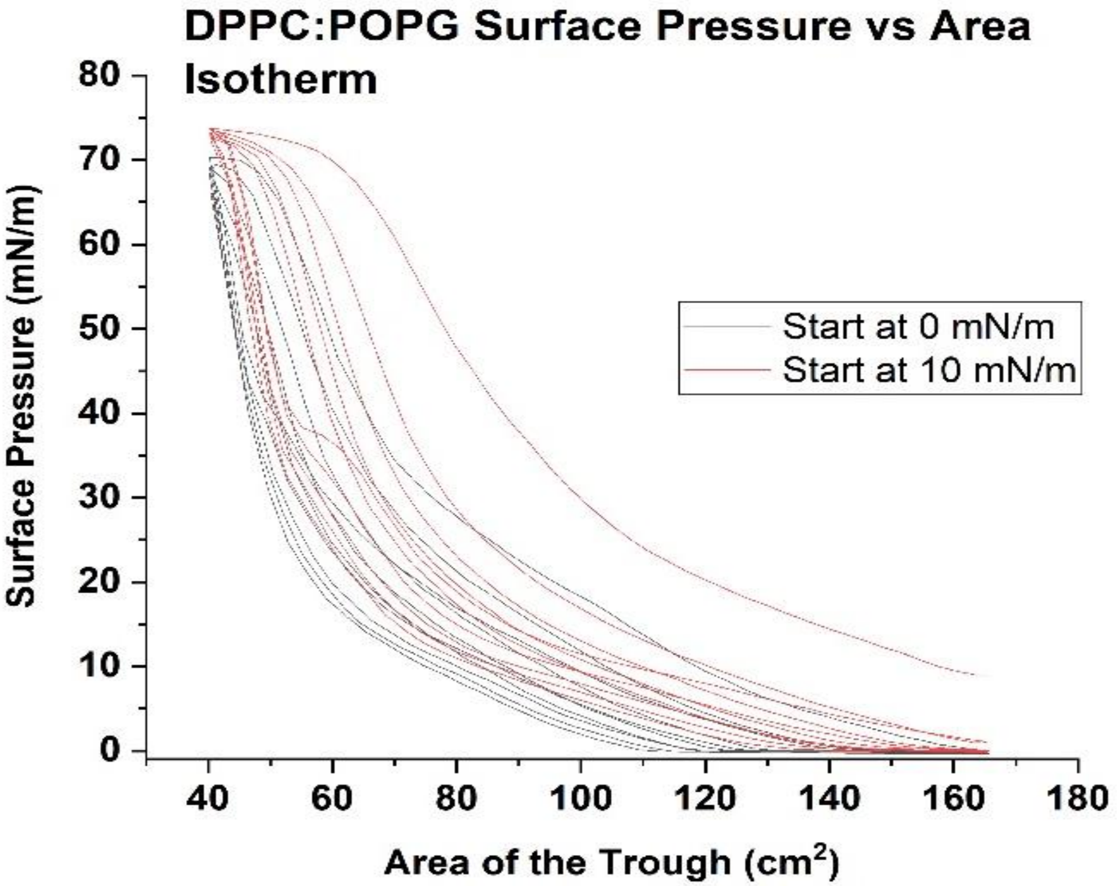

Figure S5: Surface Pressure vs. trough area isotherm for DPPC:POPG films using two different starting surface pressures.
